# Experiences and challenges of healthcare professionals in oncological surgery during the COVID-19 pandemic: A qualitative study

**DOI:** 10.1371/journal.pone.0361492

**Published:** 2026-10-27

**Authors:** Antonia L. Stengler, Elke Mattern, Ulrich Ronellenfitsch, Anke Steckelberg

**Affiliations:** 1 University Hospital Halle (Saale) and Medical Faculty of the Martin Luther University Halle-Wittenberg, Department of Visceral, Vascular and Endocrine surgery, Halle (Saale), Germany; 2 Institute of Health, Midwifery and Nursing Science, Medical Faculty, Martin Luther University Halle-Wittenberg, Halle (Saale), Germany; University of Foggia: Universita degli Studi di Foggia, ITALY

## Abstract

**Introduction:**

The COVID-19 pandemic posed significant challenges not only to healthcare systems but also to healthcare professionals. This study explores the experiences, perceptions, and perceived burdens of healthcare professionals involved in the care of patients undergoing oncological surgery during the pandemic, with a particular focus on clinical staff working in hospital-based care in Saxony-Anhalt, Germany.

**Methods:**

A qualitative, interpretive-hermeneutic approach was employed using one focus group and one individual semi-structured interview. Participants were healthcare professionals involved in the care of patients undergoing oncological surgery. The focus group included 12 participants, and one additional participant took part in an individual interview. The transcripts were independently coded and analytically annotated by two researchers and systematically analyzed in an iterative process using MAXQDA. The analysis combined deductively informed codes derived from the interview guide with inductively developed codes emerging from the data. The study was reported in accordance with the Consolidated Criteria for Reporting Qualitative Research (COREQ) reporting statement.

**Results:**

In September 2024, one focus group and one individual interview were conducted with a total of 13 participants, predominantly nurses and nursing assistants. Nine categories were identified. Participants described substantial emotional, professional, and organizational burdens during the COVID-19 pandemic, including stress, anxiety, increased demands, uncertainty, and difficulties in maintaining usual standards of patient care. Participants also reported challenges related to postponed or adapted oncological treatments, communication with patients under infection-control measures, postoperative care under capacity constraints, team collaboration, and the availability and use of personal protective equipment. Political measures, changing regulations, and the occupational vaccination mandate were perceived by participants as sources of uncertainty, frustration, and reduced autonomy. Despite these challenges, participants also described experiences of team cohesion and professional commitment. Participants expressed a need for clearer guidance, accessible contact persons, psychosocial support, supervision, and greater involvement in decision-making.

**Conclusion:**

The findings suggest that healthcare professionals involved in hospital-based oncological surgical care experienced substantial emotional, ethical, and organizational challenges during the COVID-19 pandemic. Participants’ accounts indicate that pandemic preparedness should extend beyond infection control and include clear communication, consistent guidance, psychosocial support, supervision, and adequate organizational resources. Given the small, convenience-based, and predominantly nursing sample, these findings should be interpreted as context-specific accounts rather than as representative of healthcare professionals in oncological surgery as a whole.

## 1. Introduction

The unexpected outbreak of the COVID-19 pandemic in 2020 posed significant challenges to healthcare systems worldwide [1]. To ensure care for COVID-19 patients and limit the spread of the virus, numerous medical procedures, including oncological surgeries, were postponed or canceled [2]. During the initial phase of the pandemic, an estimated 2.3 million surgical procedures were affected globally [3]. In a multicenter analysis of 66 hospitals in Germany, the number of patients undergoing general and visceral surgery decreased by 22.7% during the first year of the pandemic [4]. Such delays may negatively affect cancer outcomes, as timely surgical intervention is important for treatment success and survival. Postponements may contribute to tumor progression, potentially complicating subsequent treatment and reducing its effectiveness [5] and may also create moral distress and ethical tensions among healthcare professionals involved in treatment prioritization [6].

Beyond these procedural and ethical challenges, the COVID-19 pandemic imposed substantial psychological and organizational burdens on healthcare professionals [7,8], including concerns about personal health, constant adaptation to evolving local and national guidelines, and increased workloads. Healthcare professionals faced a complex and unpredictable environment that challenged their emotional resilience and their ability to provide patient-centered care [9]. Even under normal conditions, nursing involves significant emotional work and requires emotional competence to respond to patients’ needs. During the COVID-19 pandemic, this emotional labor intensified, with nurses experiencing a wide range of emotions that influenced both care delivery and personal stress [9].

Pandemic-related burnout among healthcare providers has been widely documented, with evidence of elevated stress, emotional exhaustion, and reduced quality of life across healthcare settings [10–12]. However, while the general psychological impact of the COVID-19 pandemic on healthcare workers is well established, research specifically focusing on the experiences of healthcare professionals involved in surgical oncology remains limited. These professionals cared for patients requiring complex oncological and perioperative treatment while simultaneously managing infection-control requirements and changing organizational conditions. Existing studies have identified stressors such as delayed procedures, resource shortages, staff reassignment, and increased administrative burdens [13,14], but provide limited insight into subjective experiences, coping strategies, and perceived burdens in the German oncological surgical context.

The oncological surgical setting warrants particular attention because patients undergoing cancer treatment may require time-sensitive interventions, complex perioperative management, continuity of care, and substantial psychosocial support [15]. During the pandemic, healthcare professionals had to balance these requirements with measures intended to reduce the risk of SARS-CoV-2 transmission. Restrictions on visitors and the use of personal protective equipment could additionally affect communication and interpersonal contact between patients and healthcare professionals. At the same time, healthcare professionals had to manage concerns about infection while addressing the complex medical and psychosocial needs of patients with cancer. In addition, Germany’s specific pandemic response, including hospital restructuring, protective regulations, and regional differences in case numbers, highlights the need for context-specific investigation [16].

A scoping review by Madi et al. identified key stressors among personnel in oncological abdominal surgery, including delayed or canceled procedures, staff reassignment, and resource constraints [13]. Similarly, Jaworska et al. reported increased advanced-stage tumor diagnoses in the context of surgical delays, as well as emotional distress during patient triage and tensions within medical teams [14]. Both studies also point to the potential of digital tools, such as telemedicine, to mitigate some of these challenges.

Although the psychological impact of COVID-19 on healthcare workers has been widely studied, evidence specifically addressing healthcare professionals involved in surgical oncology, particularly in Germany, remains limited. This study therefore aimed to explore the subjective experiences, perceptions, and perceived burdens of clinical staff involved in hospital-based oncological surgical care in Saxony-Anhalt during the COVID-19 pandemic. By focusing on participants’ accounts, the study seeks to provide context-specific insights into the emotional, ethical, organizational, and professional challenges encountered during the pandemic and into perceived support needs that may inform preparedness for future healthcare crises.

## 2. Methods

This study is reported in accordance with the Consolidated Criteria for Reporting Qualitative Research (COREQ) checklist (S1 File).

### 2.1. Study design

A qualitative study design was selected, methodologically based on an interpretive-hermeneutic approach utilizing focus groups and individual interviews. Data collection consisted of one focus group discussion and one individual semi-structured interview. The study follows a framework inspired by the hermeneutic philosophy of Gadamer, described by Fleming et al. in a five-step process [17]. In the first step, the researchers (ALS, EM) collaboratively developed the initial questions for the interview guide. The second step comprised the identification of the researchers’ pre-understandings, which stemmed from their own assumptions, experiential knowledge, and insights gained from the literature. These pre-understandings were discussed between the two researchers and documented before data collection. Based on these preconceptions, the interview guide was developed. In the third step, the researchers gained understanding through engagement in dialogue with the study participants. The fourth step involved deepening this understanding through interpretive interaction with the text. In line with the hermeneutic circle, understanding develops through movement between parts and the whole, as the whole becomes clearer, the meaning of the parts expands. In the fifth step, the trustworthiness of the research process was supported by providing a detailed, exemplary description of the procedure [17].

### 2.2. Sampling

Eligible participants included healthcare professionals from a hospital in Saxony-Anhalt, Germany, who were involved in the care of patients undergoing oncological surgery. The study population included physicians, registered nurses, nursing assistants, and medical assistants working in the fields of internal medicine, gastroenterology, surgery, general medicine, and oncology. Recruitment was conducted between April and September 2024. 35 healthcare institutions and medical practices across Saxony-Anhalt were contacted. Invitations were sent directly to senior and chief physicians, nursing managers, and medical practices and were also distributed through internal email distribution lists to relevant departments. 34 did not provide any participants. All 13 participants were recruited from one hospital and worked in two different departments. Eligibility criteria included age > 18 years, sufficient proficiency in German, and having worked during the COVID-19 pandemic (March 11, 2020–May 5, 2023) with responsibilities involving the care of patients undergoing oncological surgery. Participants were selected using convenience sampling. Participation was voluntary and no financial compensation was provided. Initial contact was made via invitation letters sent by email. Follow-up inquiries regarding participation were conducted through personal or telephone contact.

The recruitment strategy aimed to include healthcare professionals from different professional groups. In particular, efforts were made to recruit physicians; however, physician participation was difficult to obtain, primarily because of their limited availability and workload. Ultimately, the sample comprised one physician, eight registered nurses, and four nursing assistants. All participants had worked in the care of patients undergoing oncological surgery during the COVID-19 pandemic. The 13 participants were recruited from one hospital and represented different professional areas within the institution. Some participants had worked together in the same clinical setting; however, no participant had a supervisory relationship with another participant in the focus group. To protect confidentiality and reduce the risk of identifying individual participants, no further information linking individual participants to specific professional roles, departments, or interview settings is provided.

Both in-person and online interviews were offered. Face-to-face discussions were preferred because they allowed greater interactional depth through the availability of nonverbal cues, spontaneous exchanges, and a more dynamic group atmosphere [18].

One focus group discussion with 12 participants was conducted and lasted 39 minutes, followed by one individual interview with one additional participant. During the focus group, participants were able to respond to and discuss each other’s contributions, while the moderator facilitated balanced participation and followed up on relevant statements. Given the relatively short duration of the focus group, the number of participants, and the breadth of topics addressed, individual topics could not necessarily be explored in depth. The individual interview provided an additional opportunity for more detailed exploration of the study topics. Nonverbal cues were not systematically coded or analyzed as separate data; however, they were considered by the moderators as part of the interactional context during the focus group.

### 2.3. Data Collection

The interview and focus group were conducted using the interview guide (S2 File). The interviews began with broad, open-ended questions, including the general opening question, “How did you experience the pandemic?” Rather than introducing the predefined themes directly, the moderators used follow-up questions in response to participants’ statements to deepen or clarify relevant topics. Several participants contributed to individual topics, and participants in the focus group also discussed topics with each other. In the subsequent discussion, emphasis was placed on topics that had not been sufficiently addressed previously, while areas already well-covered were de-emphasized. Rather than defining thematic saturation as a formal endpoint, the researchers assessed whether the prespecified topics had been sufficiently covered and whether further data collection was likely to provide additional substantive information.

We developed a semi-structured interview guide based on the study objectives and the chosen methodological approach (interpretive-hermeneutic framework). The initial interview guide was developed based on the researchers’ pre-understandings and the relevant literature and was revised iteratively throughout the research process.

The individual interview was conducted first, followed by the focus group discussion. Following the first data collection, the interview guide was adapted to further explore topics that had not been sufficiently addressed and to incorporate topics that had emerged inductively from the participants’ accounts. During the subsequent focus group discussion, previously identified topics were explored in greater depth, while newly emerging topics were also addressed. The individual interview also contributed additional information, particularly regarding political measures and participants’ perceptions of pandemic-related regulations.

Informed consent was obtained prior to each interview and focus group discussion. The consent script was sent to interested participants via email and was also provided again immediately before the interview. Participants were given the opportunity to ask questions before signing the consent form. Demographic data were collected immediately prior to the interviews via a short questionnaire.

Data collection was considered sufficient when the topics specified in the interview guide had been addressed with sufficient breadth within the available data, while newly emerging topics had also been incorporated into the interview guide and coding framework. The researchers compared the material from the individual interview and the focus group discussion and found that the second data-collection session added further information and led to some refinement and differentiation of existing codes, but did not require the establishment of substantially new thematic areas. After the second data collection, the researchers did not identify further substantive themes that required additional exploration. No further interviews were conducted because no additional participants were available or had agreed to participate within the recruitment period and the available timeframe. Accordingly, the study does not claim formal thematic or theoretical saturation in a strict methodological sense. The researchers considered the prespecified topics sufficiently covered within the available dataset; however, they could not determine with certainty that additional interviews would not have generated new themes.

No pilot testing was conducted and no repeat interviews were carried out. All interviews were audio-recorded with participants’ permission and no field notes were taken. Participants were informed that the transcripts would be analyzed afterward. Participants were offered the opportunity to review and approve their interview transcripts, however, transcript review was not undertaken. They were also given the opportunity to ask questions or raise concerns during and after the interviews, including by email. No follow-up clarification was required because potentially unclear points were addressed during the interviews.

### 2.4. Data analysis

The individual interview and focus group discussion were transcribed verbatim immediately after each session. One of the interviewers (ALS) reviewed the audio recordings for accuracy, read the transcripts carefully, made necessary additions, and de-identified the data.

To gain an overview of the material, summaries were written for each transcript. These were followed by a detailed analysis of individual text segments. The data were independently coded and annotated by two researchers (ALS, EM).

The initial coding framework was developed deductively based on the interview guide, the study objectives, and the researchers’ pre-understandings. Four main codes with corresponding subcodes were predefined, and relevant text segments were initially assigned to these codes. During the analysis, additional subcodes were developed inductively from the interview material when emerging aspects could not be adequately captured by the existing framework. Existing codes were also modified, differentiated, or reorganized where required. Thus, the coding process combined deductive and inductive elements and evolved iteratively throughout the analysis.

ALS and EM coded and annotated the transcripts independently. Differences in coding or interpretation were discussed by returning to the relevant transcript passages and considering the respective interpretations. Where existing codes did not adequately capture a passage, new or modified codes were developed. The coding framework was revised where necessary, and consensus was reached through discussion between the two researchers.

The development of codes, subcodes, categories, and corresponding comments was documented within the MAXQDA project. The individual coding and annotations of both researchers remained separately traceable. In addition, the comments and the resulting synthesis were reviewed by two independent members of the project team and subsequently discussed and reviewed by the researchers. This additional review served to examine the consistency and plausibility of the interpretation and synthesis. Contradictory or differing accounts were retained during analysis and considered when developing the categories rather than being excluded solely because they differed from the dominant pattern.

Finally, the researchers’ collective comments within each category were synthesized into result texts, supported by illustrative quotations. As the interviews and focus group discussion were conducted in German, quotations were translated into English by ALS, with minor linguistic adaptations to preserve meaning and comprehensibility. The translations were subsequently checked independently by a bilingual reviewer against the original German transcripts to verify their meaning and tone. The original German wording was retained in the transcripts throughout the analysis. Data management was conducted using MAXQDA, version 23.

### 2.5. Reflexivity

The individual interview and focus group discussion were conducted by two female, experienced moderators (ALS, EM), both of whom hold a Master of Science degree and are employed as research associates. Both moderators had prior experience with qualitative research methods but had no specific clinical expertise in COVID-19 or surgical oncology. At the time of the study, neither moderator had a professional relationship with the participants.

Both researchers were scientifically active during the COVID-19 pandemic, but their professional work was in fields other than oncological surgical care. Neither researcher was a surgeon, nor had either worked in an outpatient clinic or hospital setting providing oncological surgical care during the pandemic. Consequently, the researchers approached the study without direct professional experience of the specific clinical setting under investigation. Before data collection, the researchers discussed and documented their pre-understandings and assumptions regarding the research topic. These pre-understandings were subsequently revisited during the analytical process to support reflexive awareness of how prior assumptions might influence interpretation.

Participants were informed about the purpose of the study through a written handout and in a personal conversation. The researchers’ personal motivations for conducting the study were not disclosed to the participants. No information about the personal or professional characteristics of the moderators was provided to the participants.

### 2.6. Ethical approval

The study received ethical approval from the Ethics Committee of the Medical Faculty of the Martin Luther University Halle-Wittenberg (Reference Number: 2023−201) and was conducted in accordance with the Declaration of Helsinki. In addition, it was approved by the staff council of the University Medicine Halle.

## 3. Results

In total, 13 individuals participated in the study. Participants’ characteristics are summarized in Table 1.

**Table 1:** Characteristics of the participants.

Demographic data	Number of participants (n = 13)
**profession** *physician* *nurse* *nursing assistant*	1 8 4
**worked during pandemic** ^**1**^ *one year or less* *two years* *entire duration of the pandemic*	3 2 8
**average professional experience**	15 years (2–41 years)
**highest educational/professional qualification** *postdoctoral qualification* *completed vocational training*	1 12
**additional private obligations** *childcare responsibilities*	2
**employed in Saxony-Anhalt**	13
**employed in a hospital setting**	13
**hospital ward** *general ward* *intensive care unit*	12 1
**average weekly working hours**	40 hours/week

A focus group discussion lasting 39 minutes and a single interview of 51 minutes were conducted.The coding process resulted in four main codes with corresponding subcodes, including additional subcodes that emerged from the interview material during the analysis. The resulting coding framework is presented in Table 2.

**Table 2:** Evolving coding framework with initial codes and inductively developed subcodes.

Codes	Subcodes
I. Changed care situation	1. Changes in daily work routine 2. Fear of making mistakes 3. Increased demands and high level of personal initiative 4. Avoidance strategies to reduce infection risk 5. Physical and psychological strain caused by hygiene measures 6. Resource scarcity 7. Personal life *
II. Psychosocially challenging situation in professional and private life	1. Postponement of diagnostics and surgeries 2. Deaths among friends, colleagues, and family 3. Changed work structure 4. Personal initiative and flexibility 5. Fear of making mistakes 6. Fear of infection 7. Not meeting personal expectations 8. Vaccination * 9. Experiences and studies * 10. Political statements *
III. Stress-related factors	1. Overload due to dynamic changes 2. Challenges with digital formats 3. Answering questions on unfamiliar topics 4. More complex patient care 5. Team dynamics *
IV. Lessons for the present and future	1. Relief: increased digital options 2. Recommendations 3. More psychosocial support for staff

* Subcodes developed inductively from the interview material and not based on the researchers’ initial assumptions.

Table 3 shows an example illustrating the assignment of a text passage to a code and subcode, along with a summarized comment based on the passage and the collaborative commentary.

**Table 3:** Transcript excerpt with corresponding code, subcode and collective comments.

Code and Subcode	Excerpt	Collective Comment
Psychosocially challenging situation in professional and private life ◊ Fear of making mistakes	*“One goes to work filled only with fear. There is fear of contracting COVID-19, fear of not being able to manage the workload, fear of failing to meet patients’ needs and fear of emergencies occurring. And there is always no one there. They are left to face it alone.”*	There is fear of not meeting patients’ needs and of being unable to manage the workload, as well as concern about not being able to reach anyone for help during emergencies.

The summarized interview material was organized into nine categories with corresponding subcategories (Table 4). The researchers’ collective comments were assigned to these categories and used to structure the presentation of the results below.

**Table 4:** Final thematic structure with identified categories and subcategories.

Category	Subcategory
I. Political measures and regulations	none
II. Emotional and professional strain	1. Failing to meet personal standards 2. Stress 3. Anxiety 4. Increased demands
III. Delays in surgeries and examinations	none
IV. Experiences and evidence	none
V. Hygiene, personal protection and safety measures	none
VI. Patient care	none
VII. Team dynamics and collaboration	none
VIII. Vaccination	none
IX. Wishes and demands	none

### 3.1 Presentation of categories

In the following section, the statements of the interview participants are presented as condensed result texts, supported by selected direct quotations and structured according to thematic categories. The findings describe participants’ reported experiences and perceptions and should not be interpreted as independently verified factual claims.

#### 3.1.1 Category I – Political measures and regulations.

Participants expressed dissatisfaction with political decisions made during the pandemic, which some perceived as insufficiently supported by available data. One participant explicitly criticized this, stating: “When I don’t have data, I say so openly: I don’t know.” (physician (P), 30 years) Participants perceived some political regulations as contributing to societal division. Some participants also expressed frustration that political decision-makers, in their view, did not communicate uncertainty sufficiently transparently, including in relation to vaccination. The political measures were considered “hysterical” and “at times poorly reflected”, which participants perceived as complicating the work of hospital staff members. Participants reported that the implementation of federal guidelines varied greatly across the federal states. Some measures were perceived as insufficiently considered and primarily reactive to the rapidly changing pandemic situation. Decisions such as contact bans or the requirement of a negative COVID-19 PCR test no older than 48 hours for the transfer of critically ill patients to another hospital were perceived by participants as arbitrary, difficult to understand, or insufficiently justified. One participant stated: “These are the kind of things I just can’t understand. Honestly, not at all.” (P, 30 years) Participants reported that these experiences contributed to a loss of trust in political authorities.

#### 3.1.2 Category II – Emotional and professional strain.

3.1.2.1 Category II.1 – Failing to meet personal standards: During the pandemic, participants reported that deceased patients with COVID-19 were placed naked in white body bags and clinical staff were prohibited from performing their customary rituals for the deceased, such as placing a flower on the body or folding their hands. Staff reported that this caused them great distress. One participant recalled: “I will never forget it. I just didn’t understand how they were treated—like a piece of dirt. I found it absolutely terrible. I was truly shocked.” (nurse (N), 41 years)

The participants reported feeling extremely frustrated that, due to protective measures, they were not even allowed to hold the hands of critically ill patients to offer the closeness and support those patients were perceived to need: “How am I supposed to hold someone’s hand when I’m wearing gloves?” (N, 32 years)

Additionally, some participants described it as a significant professional challenge when previously established approaches to treatment had to be adapted in response to changing regulations. They perceived this as conflicting with their professional principles and values.

3.1.2.2 Category II.2 – Stress: Several participants expressed concern about the spread of claims that they perceived as insufficiently supported by evidence, such as the assumption that vaccinations could reactivate cancer. Additionally, interviewees highlighted the stress caused by assertions that they perceived as having been presented with greater certainty than the available evidence warranted, including the belief that vaccinations provide complete protection against infection. These statements caused some participants significant frustration, particularly when they perceived that conclusions were not based on solid data. Existing regulations were often perceived as arbitrary prohibitions and coercion that participants felt were enforced without sufficient justification. The perceived coercive situation and the multitude of restrictions placed a heavy burden on the staff, who felt externally controlled and not granted sufficient autonomy. As one participant reported: “I have a huge problem with bans and coercion.” Our entire will was taken away from us.” (P, 30 years) Some participants described feeling compelled to focus on their professional duties and had little energy to challenge regulations they deemed pointless, because “almost every day or hour, some new nonsense was being added again.” (P, 30 years) Some employees remained uncompromising and felt bound by their ethical code, even if this entailed the risk of being sanctioned.

3.1.2.3 Category II.3 – Anxiety: Participants described the COVID-19 pandemic as a source of considerable anxiety and stress. One participant associated the described workload and stress with concerns about burnout, psychological difficulties, and anxiety. At the onset of the pandemic, the constant fear of being COVID-positive was described as pervasive by some participants. Particularly distressing was the fear of bringing the virus home and thereby endangering family members. Over time, it became common that colleagues tested positive, which intensified the feeling of being the next person to be affected. A participant recalled: “You went to work every day just waiting for the moment you'd test positive.” (N, 11 years) Fear of failing to meet patients’ needs and an inability to manage one’s workload was also prevalent: “You go to work and you're just afraid. You're afraid of getting COVID. You're afraid of not being able to handle the workload.” (nurse assistant (NA), 3 years) In emergency situations, there was concern about being unable to reach anyone for support. Participants described this additional responsibility as increasing their fear of making mistakes. The treatment teams also feared that postponements of care could lead to more urgent and difficult-to-treat conditions. Participants expressed concerns that severely ill patients could not be transferred due to overcrowded wards, as the focus shifted increasingly toward the treatment of COVID-infected patients. Nurses stated they had to collaborate with physicians whom they felt did not always adhere to hygiene protocols, raising concerns about patient safety. However, some participants reported no fears, even when they had caregiving responsibilities for children or other family members.

3.1.2.4 Category II.4 – Increased demands: The COVID-19 pandemic posed significant challenges to the healthcare system and increased demands on the treatment teams. Despite the difficult circumstances, healthcare professionals described efforts to adhere to the prescribed guidelines based on their medical knowledge and conscience and to maintain as normal a workflow as possible: “I tried to continue my work as good as I could.” (P, 30 years) Patients continued to be included in experimental treatments and clinical trials. Staff were sometimes required to put on and change PPE repeatedly throughout the day, adding to an already strenuous workday. It was reported that wearing masks caused not only skin irritations but also shortness of breath. According to a participant, daily decisions had to be made about which information was relevant, with regulations “sometimes changing hourly” (P, 30 years). Furthermore, it was perceived challenging to engage in discussions with hospital management about unclear regulations in order to achieve their repeal. For example, participants described the continuation of cancer surgery as particularly important because of the perceived time-sensitive nature of oncological conditions. One interviewee commented on this as follows: “The cancer, however, is real and poses a 100% threat right now, unlike COVID, which is still hypothetical in that moment.” (P, 30 years)

Uncertainty regarding the pandemic’s progression complicated comprehensive patient education, as no one knew exactly how the situation would develop. Initially, hospital staff were unsure how to effectively prevent infection and therefore experimented with various hygiene measures. Participants described that, over time, they became more accustomed to the implementation of hygiene measures and developed greater practical experience in dealing with them.

An additional burden arose from the fact that patients often were transferred directly to regular wards after surgery because intensive care units had to remain reserved for emergencies.

In some cases, colleagues cared for the children of other hospital staff to enable them to fulfill their professional duties. A participant recalled: “We always tried to find solutions. As I said, we were fully aware of what we were dealing with.” (P, 30 years) Due to staffing shortages, participants reported that at times asymptomatic infected nurses were required to continue working while wearing masks. When nursing staff were ill or in quarantine, some physicians took on additional tasks, rescheduled patients, and administered chemotherapy themselves. Training sessions were held online, which posed a problem because questions could sometimes not be asked in digital formats, making follow-up more time-consuming. As a result, digital options were often rejected. Nevertheless, some employees perceived the digital formats as advantageous because they functioned smoothly for them.

#### 3.1.3 Category III – Delays in surgeries and examinations.

A participant reported with the statement “come what may, surgery must go on” (N, 32 years) that during the pandemic, numerous surgeries continued to be performed. However, uncertainties regarding future lockdowns or political measures were perceived to lead to reductions and compromises in the treatment process. To enable faster and easier workflows, the execution of examinations, therapies, and surgeries was therefore adapted. Physicians had to carefully weigh benefits and risks, particularly with regard to whether cancer treatment was more urgent than the risk of potential COVID-19 infection.

#### 3.1.4 Category IV – Experiences and evidence.

One participant referred to findings from a separate study conducted during the pandemic. According to this participant, there was no overall increase in cancer-related mortality compared with previous years, however, the participant reported a delayed increase in mortality rates for gastrointestinal tumors and gynecological cancers two years later. The participant further reported that all certified cancer centers in Saxony-Anhalt had been able to maintain treatment delivery at levels comparable to those in 2018 and 2019.

Nevertheless, the participant reported that outcomes such as overall survival, disease-free intervals, and time to progression were worse. The participant attributed this decline to perceived deviations from established clinical guidelines. The rapid modification of guidelines by cancer societies without, in the participant’s view, sufficient evidence was perceived to contribute to patient uncertainty, particularly regarding vaccination recommendations.

One participant, who reported having been involved in a vaccination study, stated that the study had found no significant change in immunity levels among cancer patients after the second vaccine dose. Some participants reported memory lapses and difficulties finding words, which they themselves attributed to previous COVID-19 illness.

#### 3.1.5 Category V – Hygiene, personal protection and safety measures.

During the pandemic, the healthcare professionals were often pitied by their families and friends due to their work in hospitals. Nevertheless, they perceived the strict hygiene measures within the clinic as safer compared to, e.g., those in public transportation such as buses or trains. The staff adhered to the prescribed guidelines and wore masks continuously throughout their entire shifts. When treating COVID-19 positive patients, they wore FFP3 masks, sometimes for up to 8.5 hours, which participants perceived as potentially inconsistent with occupational safety requirements. One healthcare worker expressed concerns regarding the reliability of the masks.

Participants reported that shortages of PPE led to situations in which staff had to request masks, while homemade masks were explicitly prohibited. The existing hygiene protocols for COVID-19 infected patients were only supplemented by wearing protective goggles and caps. Participants reported that staff on infection wards were provided with inexpensive “safety glasses” that frequently slipped off and were uncomfortable when worn together with masks. Participants reported that high-quality, well-fitting goggles were considered too costly by their employers. Consequently, healthcare workers independently procured better-fitting eyewear that did not interfere with their work, but had to bear the costs themselves.

The available facilities continued to be used in accordance with pandemic regulations. The hygiene guidelines did not address footwear, which was criticized by participants. Shoes were stored in lockers after shifts and reused on the following day, raising concerns about infection control. One participant remarked: “We entered patients’ rooms wearing the same shoes we wore throughout the ward. Why were there no hygiene regulations for that? You carry germs with you, they were also on the floor. According to hygiene standards, we were spreading those germs further.” (N, 41 years) Although COVID-19 infections were reported among staff, participants perceived inadequate protective measures on the wards as one possible concern. The study did not assess the source of infection or establish whether infections were occupationally acquired.

#### 3.1.6 Category VI – Patient care.

The pandemic represented an extremely challenging and threatening situation for both the healthcare teams and the patients. Contact with patients was severely limited by the use of PPE, which was felt by nursing staff to significantly complicate their tasks and to impair patients’ trust. Furthermore, communication was felt to be hampered by mask-wearing due to reduced audibility and clarity. Participants reported that nurses only entered patient rooms when they considered it necessary or in emergency situations. One participant explained: “You only went into the room if you really had to.” (N, 7 years) There was concern that patients might be fearful not only because of their illness but also due to the pandemic.

Participants perceived it as inappropriate that some healthcare professionals contributed to feelings of guilt among cancer patients regarding vaccination, which they perceived as insufficiently supported by evidence. An interviewee reported: “Do you know what kind of uncertainty that creates? And then there are general practitioners, who tell patients all sorts of nonsense. Like: ‘Your cancer got worse because you got vaccinated.’” (P, 30 years) Care and support within patient rooms required more time than before the pandemic, particularly due to the increased need for psychosocial support. This was mainly because patients, restricted by visitation bans and lacking access to electronic communication tools, could not be contacted by phone. A participant recalled: ”We were, in a way, like family. Yes, that was something that came on top of everything else. We had to shoulder three times as much as we normally do.” (N, 11 years) Bed allocation was carried out considering infection risks and fear of contagion.

Although treatment appointments were generally not significantly prolonged, participants reported that many healthcare workers adapted their treatments to the new circumstances during the pandemic. Patient consultations continued to take place on-site and patients with life-threatening cancer diagnoses still gladly came to the clinic to continue their treatment. Participants reported that patients expressed gratitude for the opportunity to receive an appointment.

#### 3.1.7 Category VII – Team dynamics and collaboration.

During the pandemic, various conflicts arose within the teams, significantly straining collaboration. On the one hand, according to some participants, physicians did not always adhere to established guidelines, causing tensions as they were not held accountable for these deviations. As one participant explained: “Some doctors didn’t take the hygiene rules seriously. There were doctors who said a mask was enough. It was all a bit strange.” (N, 32 years) This was described as contributing to tensions between doctors and nursing staff. On the other hand, nurses reported maintaining physical distance even from their closest colleagues within the team to protect themselves from infection, which also contributed to conflicts. In one discussion, it was mentioned that the treatment team had already suffered from a lack of cohesion prior to the pandemic. There was talk of a general sense of disappointment extending beyond the challenges posed by COVID-19, linked to working conditions and the perceived appreciation of staff. However, in another conversation, it was emphasized that despite the difficulties during the pandemic, the treatment team remained united and pursued a common goal without division. This sense of solidarity instilled pride among the staff, as did the fact that all personal concerns were set aside for the benefit of patients in oncology care. The close collaboration between different disciplines and professional groups was also highlighted. An interviewee stated: “I’m proud of the teams. That we stuck together, that COVID didn’t matter to us. We still had our mission. We really put our private issues aside.” (P, 30 years)

#### 3.1.8 Category VIII – Vaccination.

Participants described the vaccination mandate as a source of conflict and emotional distress. It was perceived as unfair that staff were threatened with sanctions, including potential dismissal, if they refused vaccination. Participants expressed dissatisfaction that, despite the elevated risk of SARS-CoV-2 infection within the hospital at the onset of the pandemic, they had continued their work as normally as possible, whereas later in the pandemic they faced the prospect of potential dismissal if they chose not to comply with mandatory vaccination requirements.

Furthermore, educating staff about the vaccine based on the available data proved particularly difficult, as participants perceived the available evidence at that time as insufficient to answer all of their questions. According to participants’ accounts, staff members who did not explicitly recommend vaccination were reprimanded by the medical director and accused of opposing the vaccine. The mandate was perceived by some participants as an infringement on bodily autonomy and an attack on one’s own body. One participant reported: “It was terrible that we were forced into it. I found the pressure really awful. I mean, it’s my body. Why does my employer get to decide that I have to get vaccinated?” (NA, 3 years)

One participant reported permanent visual impairment following the first vaccination with AstraZeneca. Participants who described having been vaccinated also reported concerns about potential long-term effects. The vaccination mandate was described by some participants as conflicting with their personal principles. A participant expressed this as follows: “I felt like I had betrayed myself.” (P, 30 years)

Participants also reported that the vaccination mandate contributed to conflicts and, according to their accounts, to resignations among staff. The mandate was perceived by some participants as a form of bullying directed at unvaccinated staff, who were required to undergo daily testing before starting work. The vaccination mandate was described as burdensome for the teams, including vaccinated colleagues. Some participants perceived the available scientific evidence at the time as insufficient to justify the measure and expressed frustration about the way the mandate was implemented.

#### 3.1.9 Category IX – Wishes and demands.

There was a strong desire for supervision sessions during and after the pandemic, as a comprehensive processing of the events had not yet taken place. Participants expressed that in times of crisis, there is a need for clear guidelines and designated contact persons to support staff on the wards. Furthermore, timely procurement of PPE and the assurance of reliable COVID-19 testing were described as important needs. The wish for larger infection wards was also emphasized, as hospitals were felt to be inadequately equipped with such facilities and thus not optimally prepared for health crises. As one participant put it: “We have to rethink things. We need larger infection units so that we are prepared. And that’s what's missing.” (N, 41 years) Some participants expressed the view that physicians should adhere to the same rules as all other staff members; otherwise, sanctions should be enforced. As one participant pointedly stated: “Doctors need to learn that they are not walking disinfectants.” (N, 32 years)

One participant reported that the political response to the pandemic had not been sufficiently reflected upon in their view and called for an intensive examination of the alleged mistakes and ill-considered measures taken in order to restore trust in politics. In the future, this participant advocated avoiding prohibitions, coercion, and hysteria, as these were perceived as unsustainable solutions. Instead, the participant called for rational decisions: “Prohibitions, coercion, and hysteria should be avoided. I actually have a problem with bans. Bans never solve a problem. We have to think rationally. We also have to accept.” (P, 30 years)

## 4. Discussion

This study explored how healthcare professionals involved in hospital-based oncological surgical care experienced the COVID-19 pandemic and perceived its consequences for professional practice, patient care, and team functioning. The findings indicate that the challenges extended beyond infection prevention and increased workload. Participants described a sustained tension between maintaining time-sensitive oncological care, adapting to changing organizational requirements, and responding to the emotional and psychosocial needs of patients and colleagues. At the same time, professional commitment, adaptability, and team cohesion emerged as important resources.

A central finding was the tension between the time-sensitive nature of oncological treatment and the need to respond to an evolving infectious disease threat. Participants described situations in which surgical procedures, diagnostic processes, postoperative care, and resource allocation had to be reconsidered in response to changing infection-control requirements and available capacities. Delaying treatment may carry clinical consequences, whereas proceeding may expose patients and healthcare professionals to additional risks or compete with resources required elsewhere. Previous literature has similarly described the need to prioritize cancer surgery during the pandemic and the ethical challenges associated with such decisions [7,13]. Importantly, participants did not describe a uniform interruption of oncological treatment. Rather, continuity of cancer care was maintained through adaptation, prioritization, and changes in clinical workflows. What distinguishes the oncological surgical context is the need to maintain continuity of time-sensitive cancer treatment while simultaneously managing perioperative infection risks, postoperative capacity constraints, and the psychosocial needs of patients whose treatment could not always be postponed without potential clinical consequences [13]. One participant reported that treatment delivery in certified cancer centers in Saxony-Anhalt remained broadly comparable with previous years while describing changes in treatment sequencing and clinical decision-making. Thus, maintaining treatment volume did not necessarily imply unchanged conditions of care.

Participants’ emotional burden was closely connected to organizational and clinical uncertainty. They reported fear of infection, concerns about transmitting SARS-CoV-2 to family members, anxiety about making mistakes, and frustration about being unable to provide care according to usual professional standards. These experiences are consistent with previous research demonstrating psychological and moral burdens among healthcare workers during the pandemic [6,19]. Emotional strain was not attributed solely to workload but also to discrepancies between professional values and the care that could be provided under pandemic restrictions. Difficulties surrounding dying patients, restrictions on customary end-of-life practices, and reduced opportunities for physical reassurance illustrate how infection-control measures affected the relational dimensions of healthcare, which may be particularly relevant in oncology, where continuity, communication, and psychosocial support are integral to patient care.

Patient isolation and PPE further altered staff–patient interactions. Participants described reduced opportunities for interpersonal contact, communication difficulties while wearing masks, and an increased need for psychosocial support when relatives could not be present. These experiences correspond with previous research showing that patient isolation can affect staff–patient interactions, while PPE and distancing may affect teamwork [20,21]. In oncology settings, facial masking has also been associated with changes in communication and perceptions of empathy [22]. Thus, infection-control measures may have consequences extending beyond physical protection to relational and communicative aspects of cancer care.

Organizational adaptation was another prominent feature. Staff shortages, quarantine-related absences, changing regulations, increased workloads, and the assumption of tasks outside usual professional roles required considerable flexibility. Some participants described physicians taking on nursing-related responsibilities or rescheduling patients when nursing staff were unavailable. Although such role expansion created additional pressure, participants also interpreted these efforts as expressions of professional commitment and collective responsibility. This dual character of adaptation is important: measures intended to maintain continuity of care may simultaneously increase workload and strengthen team cohesion. Previous research has linked increased workload with poorer quality of life among nurses during the pandemic [23], while studies of healthcare teams have highlighted both disruptive and supportive effects of pandemic-related changes in working practices [21].

Team functioning was characterized by both conflict and solidarity. Differences in adherence to infection-control practices, staffing shortages, and pre-existing dissatisfaction with working conditions contributed to tensions, whereas several participants described strong solidarity and a shared commitment to maintaining oncological care. This coexistence suggests that team functioning during a crisis is shaped not only by workload or infection risk but also by existing relationships, perceptions of fairness, professional responsibilities, and organizational support. For oncological surgical services, maintaining interdisciplinary collaboration remains important because treatment depends on coordination across multiple clinical and supportive structures.

Another important dimension was trust in institutional and political decision-making. Participants described rapidly changing regulations and perceived inconsistencies in their implementation, which they associated with frustration and reduced confidence in decision-makers. The vaccination mandate was experienced by some participants as a restriction of autonomy and a source of conflict within teams. These accounts represent participants’ perceptions rather than evaluations of the scientific validity or policy justification of individual measures. The qualitative data cannot establish whether particular regulations were appropriate, whether vaccination policies were justified, or whether reported adverse events were causally attributable to vaccination. Nevertheless, participants’ accounts suggest that the communication and implementation of changing regulations may themselves shape healthcare professionals’ experiences during a crisis. Transparent communication of uncertainty and clear communication of changing requirements may therefore be important components of crisis management. This is consistent with qualitative research among healthcare workers in Germany, which identified transparent communication, clear instructions, leadership, participation, and organizational support as important factors contributing to resilience during the COVID-19 pandemic [24].These findings also point to the importance of organizational support beyond material resources, including accessible contact persons, clearer guidance, supervision, opportunities for reflection, reliable access to PPE and testing, and sufficient capacity to manage infectious patients. Organizational preparedness should therefore encompass both infection-control procedures and mechanisms enabling staff to understand changing requirements, discuss ethical and clinical uncertainties, and process difficult experiences [24,25].

Several methodological considerations are important when interpreting these findings. The study included 13 participants recruited through convenience sampling from a single hospital, with a predominantly nursing-based sample. The limited recruitment response may have contributed to self-selection and volunteer bias. The findings therefore primarily reflect nursing perspectives and should not be interpreted as representing physicians or healthcare professionals in oncological surgery more broadly. The single focus group and individual interview also constitute a limited qualitative dataset, and the relatively short focus group duration may have limited the depth with which individual topics could be explored. Additional interviews or focus groups may have generated further perspectives.

The retrospective nature of the study represents a further limitation. Participants were interviewed once in September 2024 and retrospectively reflected on experiences spanning March 2020 to May 2023; no longitudinal follow-up was conducted. This period was characterized by substantial changes in epidemiological conditions, circulating variants, vaccination coverage, testing strategies, and infection-control regulations. Consequently, findings may be affected by recall limitations and subsequent reinterpretation in light of later developments. Because the study did not systematically distinguish between pandemic phases, the findings should be understood as retrospective accounts spanning different stages rather than phase-specific comparisons. In addition, the study did not include standardized measures of mental or physical health, burnout, or workplace well-being. Accordingly, the findings describe subjective perceptions and reported experiences rather than objectively measured changes in health or well-being over time.

The absence of participant validation is another limitation. Participants were offered the opportunity to review and approve their interview transcripts, however, transcript review was not undertaken. This limited the opportunity to assess whether the researchers’ interpretations fully reflected participants’ intended meanings. Credibility was instead supported through independent coding by two researchers, iterative comparison and discussion, review of original transcript passages in cases of disagreement, documentation of coding decisions in MAXQDA, examination of differing accounts, and additional review of the analytical synthesis by members of the research team. The combination of deductive and inductive coding kept the analysis connected to the research questions while allowing emerging issues to be incorporated.

Despite these limitations, the study has several strengths. Combining a focus group with an individual interview enabled both interactional and individual perspectives. Independent coding by two researchers and consideration of contradictory accounts strengthened analytical transparency and reduced the risk of privileging dominant narratives. Most importantly, the study provides context-specific insight into how the pandemic affected oncological surgical care from the perspective of staff directly involved in its delivery.

Taken together, the findings suggest that preparedness for future healthcare crises in oncological surgery should address both clinical continuity and healthcare professionals’ working conditions. Clear communication structures, reliable organizational guidance, sufficient staffing and material resources, accessible supervision, and opportunities for professional participation in decision-making may help teams navigate situations in which clinical priorities and infection-control requirements compete. Maintaining team cohesion and supporting the relational dimensions of patient care may therefore be important components of pandemic preparedness rather than secondary concerns.

## 5. Conclusion

This qualitative study explored participants’ experiences of hospital-based oncological surgical care during the COVID-19 pandemic. Participants described uncertainty, increased responsibility, organizational constraints, emotional strain, and ethical tensions associated with maintaining cancer care under changing conditions. These included challenges related to treatment prioritization, postoperative care, patient communication, staffing, and professional collaboration.

Pandemic-related burden extended beyond workload and infection concerns. Participants also described frustration, moral conflict, perceived loss of professional autonomy, and concerns about institutional communication and trust. At the same time, professional commitment, adaptability, and team cohesion were described as important resources.

Participants’ accounts suggest that future preparedness in oncological surgical settings may benefit from adequate staffing and material resources, clear communication structures, accessible supervision, opportunities for reflection, and mechanisms for professional participation in decision-making. Crisis guidance may also need to combine clear overarching standards with sufficient flexibility for context-specific clinical judgement in time-sensitive oncological care.

These implications should be understood as context-specific insights derived from participants’ accounts rather than as generalizable evidence. Nevertheless, the findings suggest that preparedness for future healthcare crises should encompass not only infection-control measures but also organizational flexibility, professional participation, ethical reflection, psychosocial support, transparent communication, and preservation of team cohesion.

## Supporting information

S1 File COREQ Checklist. (DOCX)

S2 File Interview Guide. (DOCX)

## Abbreviations

ALS: Antonia Louisa Stengler
COREQ: Consolidated Criteria for Reporting Qualitative Research
EM: Elke Mattern
N: nurse
NA: nurse assistant
P: physician
PPE: personal protective equipment
