# Supplementary material for: Experiences and challenges of healthcare professionals in oncological surgery during the COVID-19 pandemic: A qualitative study
Source: PLoS One. 2026 Oct 27;21(10):e0361492. doi: 10.1371/journal.pone.0361492 (PMC13712669; doi:10.1371/journal.pone.0361492)
Supplement: Supplementary file 1 [file pone.0361492.s001.docx]

**Supplementary Table 1.** Consolidated criteria for Reporting Qualitative research (COREQ) Checklist

| **Domain 1: Research team and reflexivity** | | |
| --- | --- | --- |
| *Personal Characteristics* | | *Location in Manuscript, section (page number)* |
| Which author/s conducted the interview or focus group? | ALS, EM | Methods (Page 12-13) |
| What were the researcher’s credentials? E.g. PhD, MD | ALS & EM (MSc) | Methods (Page 13) |
| What was their occupation at the time of the study? | ALS & EM (Research Associate) | Methods (Page 13) |
| Was the researcher male or female? | Female | Methods (Page 13) |
| What experience or training did the researcher have? | ALS & EM (training in qualitative research methods) | Methods (Page 13) |
| *Relationship with participants* | | |
| Was a relationship established prior to study commencement? | No | Methods (Page 14) |
| What did the participants know about the researcher?  e.g. personal goals, reasons for doing the research | **No information about the personal or professional characteristics of the moderators was provided to the participants.** | Methods (Page 14) |
| What characteristics were reported about the interviewer/facilitator? e.g., Bias, assumptions, reasons and interests in the research topic | **No information about the personal or professional characteristics of the moderators was provided to the participants.** | Methods (Page 14) |
| **Domain 2: Study design** | | |
| *Theoretical framework* | | |
| What methodological orientation was stated to underpin the study? e.g. grounded theory, discourse analysis, ethnography, phenomenology, content analysis | Interpretive-hermeneutic approach according to Gadamer | Methods (Page 7) |
| *Participant Selection* | | |
| How were participants selected? e.g. purposive, convenience, consecutive, snowball | Convenience | Methods (Page 8) |
| How were participants approached? e.g. face-to-face, telephone, mail, email | Recruited via e-mail, personal, telephone | Methods (Page 8) |
| How many participants were in the study? | 13 | Results (Page 8) |

| How many people refused to participate or dropped out? Reasons? | nobody | Methods (Page 8) |
| --- | --- | --- |
| *Setting* | | |
| Where was the data collected? e.g. home, clinic, workplace | in a hospital setting | Methods (Page 8-9) |
| Was anyone else present besides the participants and researchers? | No | Methods (Page 9) |
| What are the important characteristics of the sample?  e.g. demographic data, date | Demographic data | Methods (Page 10) |
| *Data collection* | | |
| Were questions, prompts, guides provided by the authors? Was it pilot tested? | Interview guides were provided by ALS & EM and reviewed by AS  There was no pilot test. | Methods (Page 11) |
| Were repeat interviews carried out? If yes, how many? | No | Methods (Page 11) |
| Did the research use audio or visual recording to collect the data? | All semi-structured interviews were audio- recorded only | Methods (Page 11) |
| Were field notes made during and/or after the interview or focus group? | No | Methods (Page 11) |
| What was the duration of the interviews or focus group? | A focus group discussion lasting 39 minutes and a single interview of 51 minutes were conducted. | Results (Page 15) |
| Was data saturation discussed? | Yes | Methods (Page 10-11) |
| Were transcripts returned to participants for comment and/or correction? | No | Methods (Page 11,13) |
| **Domain 3: analysis and findings** | | |
| *Data analysis* | | |
| How many data coders coded the data? | Two (ALS, EM) | Methods (Page 12) |
| Did authors provide a description of the coding tree? | Yes | Methods (page 12-13), Tables 2 and 4 |
| Were themes identified in advance or derived from the data? | Yes | Methods (Page 12-13) |
| What software, if applicable, was used to manage the data? | MAXQDA Version 23 | Methods (Page 13) |
| Did participants provide feedback on the findings? | Only Interviewers reviewed results | Methods (Page 13) |
| *Reporting* | | |
| Were participant quotations presented to illustrate the themes/findings? Was each quotation identified? e.g., participant number | Yes. Quotations associated with participant number | Results (Pages 17-28), Table 3 |
| Was there consistency between the data presented and the findings? | Yes | Results (Pages 17-28), Table 2,4 |
| Were major themes clearly presented in the findings? | Yes, Nine categories are clearly presented in Table 4 and the described in the Results section. | Results (Pages 17-28) Table 4 |
| Is there a description of diverse cases or discussion of minor themes? | Yes | Results (Page 17-28) |
