# Supplementary material for: Experiences and challenges of healthcare professionals in oncological surgery during the COVID-19 pandemic: A qualitative study
Source: PLoS One. 2026 Oct 27;21(10):e0361492. doi: 10.1371/journal.pone.0361492 (PMC13712669; doi:10.1371/journal.pone.0361492)
Supplement: Supplementary file 2 [file pone.0361492.s002.docx]

**Interview Guide**

**Introduction:**

- Welcome the participants (create a pleasant and open atmosphere)
- Brief introduction to the topic of the focus group discussion
- Description of the procedure and planned duration of the focus group discussion
- Introduction of the moderators and encouragement for participants to introduce themselves
- Clarification of any open questions

**Opening question:**

The pandemic emerged very unexpectedly in spring 2020, and shortly thereafter its consequences affected virtually everyone. New infection numbers were presented daily, along with new findings and guidelines for dealing with the COVID-19 virus and with individuals infected with SARS-CoV-2. Hygiene measures and quarantines were introduced, as well as self-protection and protective measures for others. As a result, many processes had to be carried out differently than usual.

Please think back to the time of the pandemic:

How did you experience the COVID-19 pandemic in your professional daily work?

How would you describe your professional routine during the pandemic period?

**Main section:**

**1. Changed care situation**

*In encounters with oncological patients: what was different during the pandemic?*

Possible follow-up questions regarding:

General changes in healthcare provision

- Changes during the pandemic period
- Difficulties encountered
- Successful aspects
- Changes in daily work routines
- No visiting permissions, no support from relatives possible

Changes in one’s own work area

- Changes within the work team
- Hygiene measures complicating daily work, limiting examinations, and making communication more difficult
- Resource shortages (protective equipment, hygiene products, medications)
- Increased demands, need for personal initiative and flexibility

**2. Psychosocially challenging situations in private and professional life**

*Did you experience an increase in psychologically stressful situations?*

Possible follow-up questions regarding:

- Postponement of examinations, surgeries, and therapies
- Positive COVID-19 test results
- Changed work structures (separation, protective measures)

*How did this affect you?*

- Emotional burden (deaths among colleagues, patients, family members)
- Emotional burden (medical professionals being unable to meet their professional self-expectations, pandemic standards could not be adequately fulfilled)

*How did your social environment or family react to your continued work in the healthcare sector?*

Possible follow-up questions regarding:

- Being avoided
- Being admired

*How did you perceive teamwork during the pandemic? Were there any changes?*

*How was professional or interprofessional exchange?*

Possible follow-up questions regarding:

- Exchange within the team
- Cross-sector exchange

*Did you experience fears?*

Possible follow-up questions regarding:

- Fear of infection, transmission to family, friends, patients
- Concerns about one’s own mental/physical health
- Fear of making mistakes (e.g., triage)
- Fear of the future

*Were you aware of support services you could turn to in stressful situations?*

Possible follow-up questions regarding:

- Types of support services

**3. Challenges**

*What challenges did you experience?*

Possible follow-up questions regarding:

- Feeling overwhelmed
- Work motivation
- Strategies to avoid infection
- Uncertainty about the effects and course of the COVID-19 virus
- Being a point of contact for patients regarding unknown topics
- Challenges with digital formats
- More complex patient care
- Childcare
- Caregiving responsibilities

**4. Lessons for the (present and) future**

*In your opinion, what should be improved in the event of another pandemic situation?*

Possible follow-up questions regarding:

- Current emergency plans
- Relief measures: information materials, working hours, increased use of digital options
- Action recommendations
- Greater responsibility among leadership

**5. Positive experiences/aspects**

*Are there also positive experiences that you can take away from the pandemic period? Are there aspects you are proud of?*

Possible follow-up questions regarding:

- Creative solutions and strategies
- Flexibility
- Gratitude from patients
- Exchange with other hospitals, practices, and colleagues, and mutual support
- Team cohesion, solidarity, and appreciation
- Sense of the importance of work
- Acquisition of greater professional competence

**6. Wishes and demands**

*What would you wish for in the event of another pandemic?*

Possible follow-up questions regarding:

- More support
- Improved organizational processes (alternative shift models, better collaboration between different departments, improved break situations)
- Appreciation from hospital representatives
- Better interprofessional communication structures
- Better communication within the team
- Supervision
- Higher pay

**Conclusion and outlook (to be initiated 15 minutes before the end):**

- Ask whether participants have anything else they would like to add
- Ask whether there are any remaining uncertainties or questions
- Information about the next steps (possibly renewed contact for follow-up questions)
- Farewell to the participants
